# Association of Blood Lead Levels with Methylenetetrahydrofolate Reductase Polymorphisms among Chinese Pregnant Women in Wuhan City

**DOI:** 10.1371/journal.pone.0117366

**Published:** 2015-02-27

**Authors:** Wei Shen, Bin Zhang, Shuyun Liu, Hongling Wu, Xue Gu, Lingzhi Qin, Ping Tian, Yun Zeng, Linxiang Ye, Zemin Ni, Qi Wang

**Affiliations:** 1 Department of Epidemiology and Biostatistics, MOE Key Lab of Environment and Health, School of Public Health, Tongji Medical College, Huazhong University of Science and Technology, Wuhan 430030, China; 2 Wuhan Women and Children Medical Care Center, Wuhan 430016, China; 3 Women and Children Medical Center of Jiang-an District, Wuhan 430017, China; Gentofte University Hospital, DENMARK

## Abstract

**Background:**

Pregnancy is an important stimulus of bone lead release. Elevated blood lead levels (BLLs) may cause adverse pregnancy outcomes for mothers and harmful lead effects on fetuses. However, the reports about maternal BLL changes during pregnancy are conflicting to some extent. This article is to explore the variations in BLLs among pregnant women. The relationships of BLLs with methylenetetrahydrofolate reductase (MTHFR) gene C677T, A1298C, and G1793A polymorphisms, which are associated with bone resorption, were also studied. A total of 973 women, including 234, 249, and 248 women in their first, second, and third trimesters, respectively, and 242 non-pregnant women, were recruited at the Wuhan Women and Children Medical Health Center.

**Methods:**

BLLs were determined using a graphite furnace atomic absorption spectrometer. Single-nucleotide polymorphisms of MTHFR were identified with the TaqMan probe method.

**Results:**

The geometric mean (geometric standard deviation) of BLLs was 16.2 (1.78) μg/L for all participants. All the studied MTHFR alleles were in Hardy-Weinberg equilibrium. Multiple-linear regression analysis revealed the following results. Among the pregnant women, those that carried MTHFR 677CC (i.e. wild-genotype homozygote) and 1298CC (i.e. mutant-genotype homozygote) exhibited higher BLLs than those that carried 677CT/TT (standardized β = 0.074, P = 0.042) and 1298AC/AA (standardized β = 0.077, P = 0.035) when other covariates (e.g., age, no. of children, education and income, etc.) were adjusted. The BLLs of pregnant women consistently decreased during the pregnancy and these levels positively correlated with BMI (standard β = 0.086–0.096, P<0.05).

**Conclusions:**

The 1298CC mutant-type homozygote in the MTHFR gene is a risk factor for high BLLs among low-level environmental lead-exposed Chinese pregnant women, whose BLLs consistently decreased during gestation.

## Introduction

Lead exposure during pregnancy may result in adverse pregnancy outcomes such as miscarriage, stillbirth, malformation, premature birth, and low-birthweight neonates. [1]. Lead can directly enter through the placenta into the fetus, whose nervous system is particularly susceptible to lead toxicity [2]. Gluson [3] found that approximately 85%–90% of blood lead burden in newborns can be attributed to the prenatal exposure of their mothers. Prenatal lead exposure is an issue of particular concern because it may result in irreversible postnatal neurobehavioral problems and decreased intelligence until adulthood [1]. Jedryehowski et al. [4] reported that fetuses with blood lead levels (BLLs) of 4.4–69.0 μg/L can suffer from lead-induced cognitive impairment and that the degree of damage is positively associated with their BLLs.

It is well known that approximately 90%–95% of lead in the body is stored in human bones [5]. Pregnancy is a powerful stimulus for bone resorption [6,7,8,9]. Hence, the BLLs of women with high bone lead content may be elevated because of pregnancy. Considerable epidemiological evidence suggests that BLLs change during pregnancy. Moura and Goncalves Vlente [10] showed that the BLLs consistently increase during the whole gestation period. By contrast, many studies demonstrated that maternal BLLs exhibit a U-shaped pattern during pregnancy and significantly increase toward the end [11,12,13,14,15].

Methylenetetrahydrofolate reductase (MTHFR) is a central enzyme in folate metabolism. MTHFR can catalyze the irreversible conversion of 5, 10-methylenetrahydrofolate to 5-methylenetrahydrofolate, which is the methyl donor for the conversion of homocysteine (Hcy) to methoionine [16]. The three common single-nucleotide polymorphisms (SNPs) in the MTHFR gene are C677T, A1298C, and G1793A. The C677T and A1298C polymorphisms affect the activity of the MTHFR enzyme [17,18]. G1793A is less frequent than the other two SNPs, and its functional significance remains unclear [19,20]. The C677T polymorphism, which is located in the amino-terminal catalytic domain, reduces the activity of a thermolabile enzyme by 35%–50% [21]. The A1298C variant is located in the carboxy-terminal regulatory region, and lymphocytes from individuals containing the 1298CC genotype exhibit approximately 60% of the in vitro MTHFR activity of the wild type [22]. Reduced MTHFR enzyme activity slightly elevates the total homocystine (tHcy) levels in the plasma [22,23]. However, other reports demonstrated that the A1298C polymorphism does not elevate tHcy concentrations, except when present with the 677T allele in “compound heterozygotes” [24,25]. The G1793A polymorphism is located in exon 11 of the MTHFR gene and can cause an arginine-to-glutamine change at codon 594 [26]. However, the effect of the G1793A polymorphism on tHcy levels has not been clarified [27].

In vitro studies suggested that Hcy interferes with the formation of collagen cross-links, prevents fibril insolubilization, inhibits lysyl oxidase, and may delay the synthesis of more complex cross-links in collagen [28,29,30]. Hcy can modulate bone remodeling process via several known mechanisms, such as increasing osteoclast activity, decreasing osteoblast activity, and direct action of Hcy on the bone matrix; hence Hcy is an independent risk factor of bone loss and low bone mineral density (BMD) [31,32]. In addition, an increased Hcy level is a strong and independent risk factor for osteoporotic fractures in older men and women [33]. Therefore, as an important genetic factor related to Hcy levels, MTHFR gene polymorphisms may affect BMD. However, recent findings on postmenopausal women present conflicting conclusions. Several studies demonstrated that MTHFR 677TT is a risk factor for low BMD, whereas other research reported that the C677T polymorphism is not significantly related to BMD [34,35,36,37].

Considering these studies and the evidence collected, we conducted a cross-sectional study to explore the potential relationship of BLLs with MTHFR gene C677T, A1298C, and G1793A polymorphisms among pregnant and non-pregnant women in Wuhan City, China. Other socio-demographic factors related to the BLLs of women were also studied.

## Materials and Methods

### Ethics Statement

Our research has been approved by the Ethics Committee of Tongji Medical College, Huazhong University of Science and Technology (IORG0003571). All participants were given the option to receive or reject the investigation, and written informed consent was obtained from each participant prior to the research.

### Study Population and Data Collection

The participants of this study included pregnant and non-pregnant women who were randomly selected and recruited during their routine prenatal or physical examination at the Wuhan Women and Children Medical Care Center from July to October in 2012. A complete explanation of our research and the requirements for potential participants were informed via a publicity board at the beginning of the research. Women living in Wuhan for at least 5 years and without previous or present contact to occupational lead exposure were regarded as target population. Women were excluded if they had any of the following diseases: osteoporosis, hyperthyroidism, diabetes, gestational diabetes, gestational hypertension, and other else diseases that affect BLLs and bone metabolism rate, and other adverse pregnancy complications. Women with a history of acute lead poisoning were also excluded. The participants were finally enrolled using random numbers provided by a computer. All participants were generally healthy. The included pregnant women had single pregnancies and were in their first (i.e., gestation of less than 12 weeks), second (i.e., gestation of 13 to 27 weeks), or third (i.e., gestation of 28 weeks until delivery) trimester.

A total of 1,000 participants, including 250 pregnant women in the first, second, and third trimesters and 250 non-pregnant women, were enrolled in the study. However, 27 participants were excluded because of their inability to provide blood samples. Finally, 973 eligible participants (i.e., 234, 249, and 248 pregnant women in the first, second, and third trimesters, respectively, and 242 non-pregnant women) were included in the research.

Questionnaires were used to collect the following socio-demographic characteristics: (e.g. age (≤ 25 years-ref., 26–35 years, > 35 years); race (Han-ref., Minority); education (≤ 12 years-ref., > 12 years); marital status (married-ref., unmarried); employment status (employed-ref., unemployed); income (≤ 5000R MB per month per capita-ref., > 5000 RMB per month per capita), current BMI (< 25-ref., ≥ 25); reproductive history [e.g. no. of child (no child-ref., ≥ 1 child) and previous pregnancies (zero-ref., once, ≥ 2 times)]; intake of prescription folate, Ca, Fe, and Zn product according to doctoral prescription (for pregnant women only, yes-ref., no); and status of passive smoking (no-ref., yes). No participant reported active smoking. All questionnaires were completed by each participant on their own with or without the help of trained investigators. The investigator immediately collected the completed questionnaires.

### Blood Sample Collection and Lead Measurement

Venous whole blood samples were collected from all participants with 5ml of vacuum EDTA anticoagulant blood tubes. All blood samples were stored at −80°C until laboratory analysis. Lead concentration in whole blood was measured using a graphite furnace atomic absorption spectrometer (SpctrAA-240FS, Varian Inc., USA). The lead standard (GBW08619) was purchased from the National Institute of Metrology, China. BLLs were expressed in μg/L. The detection limit of BLLs was 0.5μg/L, and no participant exhibited BLL values lower than the detection limit. All samples were measured in duplicates. The relative standard deviation (RSD) of the precision test was 3.9%–5.7% (*n* = 7), and the recovery of standard addition was 90%–105%.

### DNA Extraction and Genotyping Assay

DNA was extracted from blood cells by using a genomic DNA extraction kit (TIANGEN Biotech Co. Ltd., Beijing, China). The extraction yielded an average of 20–40 μg DNA/ml whole blood. The extracted DNA was quantified with a nucleotide analyzer (ND-1000, Thermo Fisher Scientific Inc., USA). The DNA samples were adjusted with Tris-EDTA buffer, partitioned into samples, and then stored at −80°C until genotyping. The C677T (rs1801133), A1298C (rs1801131), and G1793A (rs4846049) SNPs of the MTHFRC gene were genotyped with a TaqMan platform and an ABI-7900 real-time PCR instrument (ABI Company, USA). For quality assurance, 10% of random sample was re-analyzed and checked for discrepancies. In this study, all samples with adequate blood volume were successfully genotyped.

### Statistical Analysis

EpiData 3.0 was used to manage all data. Data analysis was conducted using SPSS (version 17.0). Statistical significance was considered at *P* < 0.05. BLLs were not normally distributed (by using the Kolmogorov–Smirnov test) and were logarithmically transformed and presented through geometric mean (GM) and geometric standard deviation (GSD). Generally, chi-square test was used to analyze enumeration data, whereas one-way ANOVA and non-parametric tests (e.g., Kruscal-Wallis H test and Mann-Whitney U test in case of heterogeneity of variance) were used to compare the BLLs of various groups. Multiple-linear regression modeling with backward selection was also performed to explore the potential relationship of pregnant BLLs to the studied MTHFR SNPs and to other related factors, such as socio-demographics and reproduction history. In the multiple-linear regression analysis, the logarithmically transformed BLLs were regarded as dependent variables. All the data used for present statistical analyses are showed in S1 Table.

## Results

### Socio-Demographic Characteristics and BLLs of All Participants

The general description of the socio-demographic characteristics and BLLs of all participants are shown in Table 1. A total of 973 participants were finally included in this research; 27 participants were excluded because of their inability to provide blood samples. The studied women were aged 17–41 years with an arithmetic mean (SD) of 27.80 (3.54) years. The mean (SD) age of pregnant women was 27.8 (3.55) years, which was not significantly different from the counterpart value of 27.6 (3.54) years among non-pregnant women (*t* = 0.837, *P* = 0.403). Most of the participants (about 99.0%) are Han people. The frequency of educational background was not significantly different between pregnant and non-pregnant women. Most of the participants (about 96.6%) are married. About 90.4% pregnant women had already given birth to their first child. About 35.8% pregnant women and 50.4% non-pregnant women had at least one previous pregnancy.

**Table 1:** Socio-demographic characteristics (*n*) and blood lead levels of all participants.

Variable		Pregnant women			Non-pregnant women	Statistics
		First trimester	Second trimester	Third trimester		
N		234	249	248	242	
BLL (μg/L, GM ± GSD)		19.3 ± 1.64	13.6 ± 1.73	12.9 ± 1.65	20.7 ± 1.82	*χ* ^*2*^ = 139.565, *P*<0.001
Age (years, mean±SD)		27.4 ± 3.35	28.0 ± 3.67	28.4 ± 3.38	27.6 ± 3.54	*F* = 5.938, *P* = 0.001
Race	Han	232	243	248	240	*χ* ^*2*^ = 6.553, *P =* 0.057^1^
	Minority	2	6	0	2	
Education (years)	≤ 12	80	88	65	181	*χ* ^*2*^ = 6.925, *P =* 0.074
	> 12	154	161	183	215	
Marital status	Married	218	249	246	227	*χ* ^*2*^ = 28.099, *P*<0.001
	Unmarried	16	0	2	15	
Employment	Employed	143	157	192	151	*χ* ^*2*^ = 19.289, *P*<0.001
	Unemployed	91	92	56	91	
Income (RMB per month per capita)	≤ 5000	157	177	146	172	*χ* ^*2*^ = 11.110, *P* = 0.011
	> 5000	77	72	102	70	
Current BMI (kg/m^2^, mean ± SD)		20.3 ± 2.72	21.6 ± 2.95	24.6 ± 3.50	20.3 ± 2.28	*χ* ^*2*^ = 251.485, *P*<0.001
No. of previous pregnancies		0	155	166	148	120	*χ* ^*2*^ = 19.986, *P* = 0.003	
		1	55	56	64	80		
		≥2	24	27	36	42		
No. of children	0	212	223	226	205	*χ* ^*2*^ = 6.387, *P* = 0.094
	1	22	26	22	37	
Hair dye (average time within six months)	Once or more	4	4	11	11	*χ* ^*2*^ = 11.265, *P* = 0.071
	0.5	119	114	111	128	
	Extremely not	111	131	126	103	
Passive smoking	Yes	27	46	30	66	*χ* ^*2*^ = 27.096, *P*<0.001
	No	207	203	218	176	
Cosmetic using	Everyday	12	5	9	66	*χ* ^*2*^ = 207.405, *P*<0.001
	Sometimes	60	53	41	104	
	Extremely not	162	191	198	72	
Prescription folate^2^	Yes	128	225	226	/	*χ* ^*2*^ = 207.405, *P*<0.001
	No	106	24	22	/	
Prescription Ca^2^	Yes	70	133	155	/	*χ* ^*2*^ = 54.136, *P*<0.001
	No	164	116	93	/	
Prescription Fe^2^	Yes	44	129	140	/	*χ* ^*2*^ = 82.161, *P*<0.001
	No	190	120	108	/	
Prescription Zn^2^	Yes	73	93	137	/	*χ* ^*2*^ = 51.758, *P*<0.001
	No	161	156	111	/	

^1^ Fisher exact chi-square test was used.

^2^ Information was obtained from pregnant women only.

The BLLs were ranged 0.39–79.83 μg/L among all participants. The GM and GSD of the BLLs were 16.2 μg/L and 1.78 μg/L for all participants, respectively. The GMs (GSDs) of the BLLs were 15.0 (1.73) and 20.7 (1.82) μg/L for pregnant and non-pregnant women, respectively; their differences were significant (*t* = 7.40, *P* < 0.001). The pregnant women in the first trimester exhibited higher BLLs than those in the second (*Z* = 7.568, *P* < 0.001,) and third (*Z* = 9.124, *P* < 0.001) trimesters. With other covariates not adjusted, the BLLs were significantly lower in the pregnant women of second (*Z* = 7.594, *P* < 0.001) and third (*Z* = 8.825, *P* < 0.001) trimesters than in the non-pregnant women. No significant differences in BLLs were observed between the pregnant women of first trimester and non-pregnant women (*Z* = 1.211, *P* = 0.226) as well as between the pregnant women of the second and third trimesters (*Z* = 1.554, *P* = 0.120).

### Distribution of MTHFR Gene Polymorphisms among all Participants

The frequencies of the C677T, A1298C, and G1793A polymorphisms of the MTHFR gene among the pregnant and non-pregnant participants are shown in Table 2. Accordingly, the frequencies (percentages) of these MTHFR gene polymorphisms were as follows: (1) 677CC (65.3%), 677CT (31.4%), and 677TT (3.3%); (2) 1298AA (35.1%), 1298AC (49.6%), and 1298CC (15.2%); and (3) 1793GG (76.2%), 1793GA (20.6%), and 1793AA (3.2%). No significant differences in the MTHFR gene polymorphisms were found between the pregnant and non-pregnant women. All the studied MTHFR alleles were in Hardy-Weinberg equilibrium.

**Table 2:** Frequencies (%) of the C677T, A1298C, and G1793A polymorphisms of the MTHFR gene among pregnant and non-pregnant participants.

MTHFR gene genotypes		Pregnant women			Non-pregnant women	Total	Statistics
		First trimester	Second trimester	Third trimester			
C677T	CC	149 (63.7)	152 (61.1)	162 (65.3)	172 (71.1)	635 (65.3)	*χ* ^*2*^ = 6.614, *P* = 0.358
	CT	75 (32.0)	89 (35.7)	78 (31.5)	64 (26.4)	306 (31.4)	
	TT	10 (4.3)	8 (3.2)	8 (3.2)	6 (2.5)	32 (3.3)	
A1298C	AA	76 (32.5)	91 (36.5)	98 (39.5)	77 (31.8)	342 (35.1)	*χ* ^*2*^ = 6.135, *P* = 0.408
	AC	122 (52.1)	124 (49.8)	117 (47.2)	120 (49.6)	483 (49.6)	
	CC	36 (15.4)	34 (13.7)	33 (13.3)	45 (18.6)	148 (15.2)	
G1793A	GG	173 (73.9)	195 (78.3)	200 (80.6)	174 (71.9)	742 (76.2)	*χ* ^*2*^ = 16.204, *P* = 0.013
	GA	56 (23.9)	48 (29.3)	44 (17.7)	52 (21.5)	200 (20.6)	
	AA	5 (2.2)	6 (2.4)	4 (1.7)	16 (6.6)	31 (3.2)	

The BLLs classified on the basis of the three MTHFR gene SNPs for all participants are shown in Table 3. No significant differences in BLLs were observed between the pregnant and non-pregnant women carrying various MTHFR gene SNPs.

**Table 3:** Blood lead levels (GM ± GSD) of pregnant and non-pregnant participants grouped according to MTHFR gene polymorphisms.

Variable		Pregnant women				Non-pregnant women
		First trimester	Second trimester	Third trimester	Total	
C677T	CC	20.0±1.72	13.8±1.74	13.4±1.63	15.4±1.75	20.5±1.81
	CT	18.2±1.51	13.2±1.73	12.4±1.62	14.3±1.67	20.1±1.88
	TT	19.0±1.54	16.2±1.39	9.5±2.21	14.6±1.83	33.2±1.42
F		0.940	0.626	2.107	1.441	1.971
P		0.392	0.536	0.124	0.237	0.142
A1298C	AA	18.8±1.50	13.2±1.74	12.6±1.73	14.4±1.72	22.0±1.77
	AC	19.3±1.74	13.6±1.71	12.8±1.59	15.0±1.73	20.3±1.80
	CC	20.5±1.59	14.7±1.78	14.4±1.64	16.4±1.71	19.4±1.98
F		0.343	0.477	0.908	2.174	0.732
P		0.710	0.621	0.405	0.114	0.482
G1793A	GG	19.8±1.72	13.7±1.74	13.3±1.63	15.2±1.75	20.5±1.82
	GA	18.2±1.49	13.2±1.73	12.3±1.62	14.4±1.66	20.2±1.81
	AA	21.6±1.65	17.7±1.38	10.8±2.17	15.6±1.93	26.6±1.92
F		0.117	0.651	0.074^1^	0.215	0.132^1^
P		0.889	0.522	0.964	0.807	0.936

^1^Non-parametric method of Kruskal-Wallis H test was used because of unequal variance, and the value of *χ* ^*2*^ was presented.

### Relationships of BLLs with MTHFR Gene Polymorphisms and other Related Factors

To further reveal the potential relationship of BLLs to the studied MTHFR gene polymorphisms and to other related factors, we conducted multiple-linear regression analysis. The results are presented in Tables 4–6. Table 4 illustrates the regression results of the factors related to the BLLs all participants. The BLLs were not associated with the studied MTHFR SNPs among all participants. Compared with the MTHFR 677CT/TT carriers, the MTHFR 677CC mutant-type homozygote carriers exhibited higher BLLs during pregnancy (standardized *β* = 0.074, *P* = 0.042). The results are shown in Table 5. The MTHFR 1298CC mutant-type homozygote carriers were more susceptible to high BLLs during pregnancy than the 1298AA/AC carriers, with a standardized *β* of 0.077 (*P* = 0.035). Other factors, including no. of children, education, income, current BMI, gestational trimester, and age, also affected the BLLs of the women during pregnancy. After controlling other covariates, the pregnant women in the second and third trimesters demonstrated lower BLLs than the non-pregnant women (both *P* < 0.001). The BLLs of the pregnant women increased and peaked in the first trimester and then decreased in the second and third trimesters. The BLLs positively correlated with the current BMI (standard *β* of approximately 0.086–0.096, *P* < 0.05). The pregnant women with low socio-economic status (SES) were susceptible to accumulating higher BLLs. The pregnant women who gave birth to at least one child had lower BLLs than those who did not (standard *β* of approximately 0.106–0.108, *P* < 0.05 for all participants and the pregnant subgroup).

**Table 4:** Results of multiple-linear regression analysis on the factors associated with the blood lead levels of all participants. ^1^

Variable	β	Standard error	Standardized *β*	t	P
Constant	2.930	0.070		41.780	<0.001
No. of children	-0.115	0.060	-0.062	1.926	0.054
Education	-0.088	0.041	-0.070	2.173	0.030
Current BMI	0.132	0.054	0.086	2.470	0.015
Second trimester of pregnancy	-0.395	0.045	-0.306	8.832	<0.001
Third trimester of pregnancy	-0.480	0.048	-0.375	9.967	<0.001

^1^ Statistics for the regression model are as follows: *F* = 27.046, *P* < 0.001 (ANOVA); *R* = 0.369, *R*-square = 0.136, adjusted *R*-square = 0.131, Durbin-Watson = 1.447.

**Table 5:** Results of multiple-linear regression analysis on the relationship of blood lead levels with the MTHFR C677T polymorphism and other related factors during pregnancy. ^1^

Variable	β	Standard error	Standardized *β*	t	P
Constant	2.821	0.082		34.615	<0.001
No. of children	-0.199	0.071	-0.108	2.826	0.005
Current BMI	0.130	0.053	0.096	2.425	0.016
Education	-0.130	0.047	-0.110	2.796	0.005
Income	-0.097	0.043	-0.085	2.242	0.025
Second trimester of pregnancy	-0.358	0.051	-0.315	7.060	<0.001
Third trimester of pregnancy	-0.451	0.053	-0.398	8.468	<0.001
MTHFR 677CC	0.084	0.041	0.074	2.038	0.042
Age of 26 to 35 years	0.119	0.050	0.096	2.393	0.017
Age of over 35 years	0.253	0.119	0.085	2.123	0.034

^1^ Statistics for the regression model are as follows: *F* = 12.058, *P* < 0.001 (ANOVA); *R* = 0.377, *R*-square = 0.142, adjusted *R*-square = 0.130, Durbin-Watson = 1.550.

**Table 6:** Results of multiple-linear regression analysis on the relationship of blood lead levels with the MTHFR A1298C polymorphism and other related factors during pregnancy.^1^

Variable	β	Standard error	Standardized *β*	t	P
Constant	2.862	0.078		36.641	<0.001
No. of children	-0.196	0.070	-0.106	2.775	0.006
Current BMI	0.122	0.053	0.091	2.288	0.022
Education	-0.132	0.047	-0.111	2.830	0.005
Income	-0.098	0.043	-0.086	2.269	0.024
Second trimester of pregnancy	-0.357	0.051	-0.314	7.038	<0.001
Third trimester of pregnancy	-0.444	0.053	-0.392	8.347	<0.001
MTHFR 1298CC	0.123	0.058	0.077	2.115	0.035
Age of 26 to 35 years	0.121	0.050	0.098	2.440	0.015
Age of over 35 years	0.266	0.119	0.089	2.228	0.026

^1^ Statistics for the regression model are as follows: *F* = 12.099, *P* < 0.001 (ANOVA); *R* = 0.377, *R*-square = 0.142, adjusted *R*-square = 0.131, Durbin-Watson = 1.568.

## Discussion

All participants exhibited BLLs lower than the level of concern (i.e., 100μg/L) recommended by the U.S. CDC in 1991 [38]. With the cessation of lead-based gasoline, the BLLs of the Chinese population significantly decrease [39]. Other reports showed that the mean BLLs of pregnant women decrease in Wuhan [40] and are lower than the reported mean (i.e., 51.3μg/L) of the Chinese population in 2001–2011[41]. In the present study, the BLLs of the pregnant women were lower than those of non-pregnant women. According to the results of multiple-linear regression analysis, the BLLs were increased and peaked in the first trimester of pregnancy and then decreased in the second and third trimesters. Our findings are similar to the results of Tellez-Rojo et al. [42] but differ from those of other studies, which demonstrated a gradually increasing trend or U-shape distribution of BLLs during pregnancy [10, 43]. Tellez-Rojo et al. [42] found that pregnant women with low bone lead and bone resorption show decreased lead levels in the plasma and blood during pregnancy. Their results suggest that the small amount of lead released from the bone of women with low lead burden and bone mobilization is compensated by the increase in plasma volume, thereby apparently reducing BLLs. By contrast, the lead levels in the plasma and blood of women with high bone lead burden and bone resorption increase during pregnancy; the high contribution of bone lead release results in a higher possibility to overcome 45%–50% increase in plasma volume [42,44]. Therefore, we speculated that both bone lead release and plasma increase during pregnancy are the principal causes of the changes in blood lead among the environmentally lead-exposed pregnant women in Wuhan, China.

Considering the present evidence that MTHFR gene polymorphisms are related to bone mineral loss, which is an important endogenous lead source during pregnancy, we assumed that MTHFR gene polymorphisms are potentially related to the BLLs of pregnant women. Our research revealed that the frequencies (percentages) of the MTHFR gene C677T, A1298C, and G1793A polymorphisms were in Hardy-Weinberg equilibrium and were similar to the reported frequencies of the three common SNPs in the MTHFR gene [17,18,19,20,32,45]. In this article, the BLLs of the pregnant women were associated with MTHFR A1298C and C677T polymorphisms but not with the MTHFR G1793A polymorphism. After adjusting other covariates, such as age, education, income, and BMI, the 1298CC mutant-type homozygote carriers possibly exhibited higher BLLs than the 1298AC/AA carriers among the pregnant women. The relationship of BLLs with A1298C polymorphism may be explained by the reduced MTHFR enzyme activity and the consequential elevated Hcy. Chia et al. [46] and Schafer et al. [47] reported that tHcy is positively associated with BLLs among lead-exposed participants in Vietnam and Singapore as well as among general old adults in America, respectively. However the 677CC carriers whose MTHFR enzyme activity levels were supposed to be higher than the 677CT/TT carriers exhibited significantly higher BLLs among the studied women. This finding should be further investigated. The MTHFR C677T polymorphism is associated with a slight increase in tHcy [48,49], but participants with the TT genotype exhibit normal tHcy if their folate status is optimal [50]. Thus far, the effect of the MTHFR haplotypes on tHcy concentrations has not been extensively studied.

The interactions of MTHFR gene polymorphisms with folate intake were also explored, but no significant results were obtained. The un-quantified folate in the participants’ plasma restricted the analysis.

In this study, the BLLs of pregnant and non-pregnant women were also associated with other factors, such as age, education, income, current BMI, and no. of children. Our findings suggest that the women with low SES are susceptible to lead exposure as confirmed by other reports [1]. Bone lead obliterated by pregnancy- and/or lactation-stimulated bone lead resorptioin may be the possible reason for the comparably lower BLLs among women who gave birth to at least one child [51,52,53,54]. Moreover, BLLs positively correlated with current BMI among the studied women, which was also confirmed by Jedrychowski et al. [55] and Polanska et al.[56]. This positive association may be determined by the total caloric or dietary fat intake. Based on a study performed on preschool children, Lucas et al. [57] speculated that the bile secreted into the gastrointestinal tract to aid in the digestion and absorption of fat may increase lead absorption.

The present research has several main limitations. First, our study was based on a cross-sectional design rather than a prospective cohort. The pregnant women in the three trimesters were randomly recruited. The non-pregnant women were also collected as a representative of the non-pregnancy controls. However, only the female residents living in Wuhan City for at least 5 years were considered in our study. These residents were similar in diet construct and living environment. Therefore, we assumed that the pregnant women and non-pregnant women were comparable. Moreover, the statistical method of multiple-linear regression analysis can be performed to reveal the relationships of the determined factors and BLLs with controlled potential covariates. Second, the sample size of our study was not large. Third, all participants were from Wuhan and were exposed to low-level environmental lead. Prudence is required when applying the conclusions from our research to different study populations. Future studies with a large sample size and based on cohort population with lead exposure at various levels should be performed to further determine the association of maternal BLLs with MTHFR gene polymorphisms and with other factors during pregnancy.

## Conclusions

The BLLs of pregnant women exposed to low-level environmental lead are associated with the C677T and A1298C polymorphisms of the MTHFR gene. The MTHFR 677CC (i.e., wild-genotype homozygote) and 1298CC (i.e., mutant-genotype homozygote) carriers are more susceptible to elevated blood lead levels than the 677TT/CT and 1298AA/AC carriers. Apparently, the BLLs of pregnant women in the second and third trimesters consistently decrease. Furthermore, pregnant women with low socio-economic status are more susceptible to acquire high BLLs.

## Supporting Information

S1 Table All the data used for the statistical analyses in this article. (SAV)
